# Multisystem Involvement Induced by Human Parvovirus B19 Infection in a Non-immunosuppressed Adult: A Case Report

**DOI:** 10.3389/fmed.2022.808205

**Published:** 2022-04-12

**Authors:** Qihang Zou, Peisong Chen, Jiaxin Chen, Dingbang Chen, Han Xia, Ling Chen, Huiyu Feng, Li Feng

**Affiliations:** ^1^Department of Neurology, The First Affiliated Hospital, Sun Yat-sen University, Guangzhou, China; ^2^Guangdong Provincial Key Laboratory of Diagnosis and Treatment of Major Neurological Diseases, National Key Clinical Department and Key Discipline of Neurology, Guangzhou, China; ^3^Department of Laboratory Medicine, The First Affiliated Hospital, Sun Yat-sen University, Guangzhou, China; ^4^Department of Scientific Affairs, Hugobiotech Co., Ltd., Beijing, China

**Keywords:** human parvovirus B19, immunosuppressed, multiple organ dysfunction, case report, mNGS

## Abstract

**Background:**

Human parvovirus B19 (B19V) infection is usually symptomless and occurs in the childhood. While in immunocompromised adults, B19V infection also presents various clinical symptoms due to the host's immune status. The classic symptoms include erythema, anemia, arthropathy, and edema, but neurological involvement is rare.

**Case Presentation:**

In this report, we present a case of B19V infection caused multiple organ dysfunction in a non-immunosuppressed adult. Metagenomic next-generation sequencing (mNGS) was used and successfully detected the pathogen in multiple types of samples, including blood, cerebrospinal fluid (CSF), and bronchoalveolar lavage fluid (BALF). The diagnosis was subsequently confirmed by polymerase chain reaction (PCR). He was treated with intravenous gamma globulin, resulting in a significant resolution of symptoms after 1 month.

**Conclusion:**

Multisystem involvement induced by B19V infection was found in this case report. mNGS performed great advantages in rapidly and accurately diagnosing B19V infection in multiple types of samples, which helps the timely adjustment of treatment and improves the prognosis.

## Introduction

Human parvovirus B19 (B19V) was first detected in 1974 (1). The most common symptom is erythema infectiosum. It is a mild exanthematous disease commonly on the face and is named “slapped cheek syndrome” due to the shape and location of the rash. In addition, the trunk and skin rashes may appear as lace-like, reticular, and spontaneously resolving macular rashes (2). Its transmission route is mainly through the respiratory tract, but it can also be transmitted by blood or from mother-to-child vertical transmission (3). Children are the main target of B19V infection. Notably, in infants, children, and adolescents with unexplained meningoencephalitis, the detection rate of B19V can be as high as 4.3% (4, 5), and the possibility of B19V pathogenesis in these cases cannot be ruled out. Adults infected by B19V can present a variety of clinical symptoms, which depend mainly on the host's immune status. In immunosuppressed adults, it can cause severe or chronic anemia due to persistent infection (6–8). The virus mainly attacks red blood cells in the hematologic system and manifests, resulting in clinical manifestations, such as rapidly worsening anemia and peripheral reticulocytopenia. Still, there are also cases with leukopenia or thrombocytopenia (37.5 and 21%, respectively) (9). In immunocompetent adults, B19V can lead to acute arthropathy (10, 11) and various neurological diseases such as encephalitis, meningoencephalitis, and Guillain-Barre syndrome (12). Alternatively, B19V has also been associated with myocarditis (13, 14) and hepatitis (15), as well as fetal hydrops and fetal abortion during pregnancy (16). In sickle cell anemia patients, B19V infection can cause transient aplastic crises.

Although B19V has previously been reported to cause various clinical symptoms in non-immunosuppressed patients, cases of B19V infection involving more than two systems are infrequent. Here we report a case who was hospitalized due to multiple organ dysfunction (MODS) caused by unexplained sepsis, without immunosuppressive background and clear focus of infection. B19V was detected in multiple samples throughout the body, including blood, cerebrospinal fluid (CSF), pleural fluid, and bronchoalveolar lavage fluid (BALF). The condition improved after blood transfusion and gamma globulin pulse therapy.

## Case Report

A 54-year-old man was admitted to the Department of Intensive Care Medicine of the First Affiliated Hospital, Sun Yat-sen University, on June 30th, 2021, due to headache and fatigue for 2 days.

The patient developed a headache on June 28th, 2021, without obvious inducement, and gradually had drowsiness, accompanied by fatigue, anorexia, and occasional cough, which could not be relieved after rest. No other abnormal symptoms were detected. The patient had a history of hypertension and gout for more than 10 years. He since had been diagnosed with sepsis and multiple organ failure in the ICU of a local hospital due to fever with upper abdominal pain. Metagenomic next-generation sequencing (mNGS) of blood on May 8th showed negative. After treatment of mepem 1 g every 6 h (Q6H), the patient was cured and was discharged 2 months ago. He did not take immunosuppressive drugs regularly for a long time and had no history of exposure to poisons, chemical agents, or animals. Personal and family histories were unremarkable.

**Figure 1:**
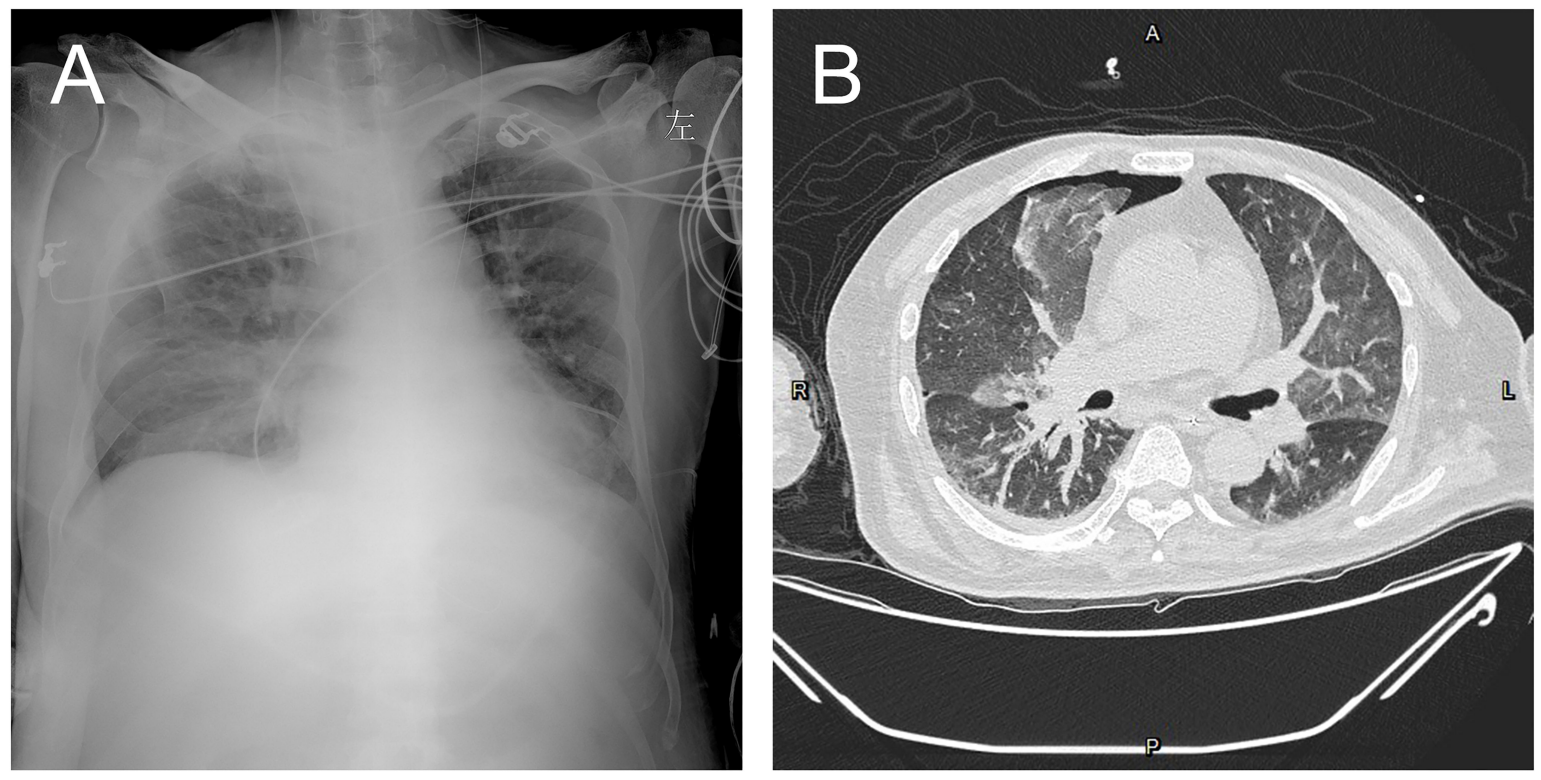
The patients' chest X-ray on July 1st **(A)** and CT on July 16th **(B)**.

Physical examination on admission showed body temperature of 36.0°C, pulse at 94 beats/min, respiratory rate of 14 breaths/min, blood pressure at 69/37 mmHg, drowsiness, chemosis, coarse breath sounds, and a small number of moist rales heard in both lower lungs. Cardio abdominal physical examination was unremarkable. Extremities were moderate edema. The movement of both upper limbs was good, and the movement of both lower limbs was limited. Auxiliary examination was also performed. Blood gas analysis showed metabolic acidosis, pH of 7.25, BE −16 mmol/L, and oxygenation >300. Elevated infection indicators analysis showed CRP of 234.75 mg/L, PCT of 5.06 ng/mL, and WBC of 41.84 × 10^9^/L. The patient was detected with mild anemia (RBC of 3.26 × 10^12^/L and Hb 93 g/L), prolonged coagulation time (APTT of 58.2 s), renal insufficiency (urea of 19.5 mmol/L and CREA 357 μmol/L), and acute heart failure (TnT-T of 0.130 ng/mL and NT-proBNP of 20813.0 pg/mL). In addition, the patient had elevated liver metabolic total bilirubin (TBIL of 79.6 μmol/L) and predominantly elevated direct bilirubin (DBIL of 55.9 μmol/L). Immunosuppressive status revealed a lower immunoglobulin M (0.49 g/L) and immunoglobulin G (7.37 g/L), B lymphocytes (CD3-CD19 +) % of 0.48%, B lymphocytes (CD3-CD19 +) count of 2.41 cells/μl, regulatory T-cells (Treg) of 18.75%, interleukin 6 of 1508.65 pg/mL, and interleukin of 10 5.57 pg/mL. Bacteriology, mycology, and tuberculosis tests of blood, sputum, urine, and stool showed no pathogens. HIV, syphilis, and Hepatitis virus tests were negative. Radiography of the pulmonary showed worsening infiltrate (Figure 1). Septic shock was considered. The patient was given Sulperazone 3 g, Q8H for anti-infection, fluid infusion, correction of acidosis, acid suppression, stomach protection, and prothrombin complex supplement on June 30th. After 7-day treatment, the infection indicators were significantly lower than before, with PCT of 1.19 ng/mL, CRP of 49.41 mg/L, WBC of 8.08 × 109/L, NEUT of 6.94 × 109/L. The conditions were under control.

On July 7th, the patient had a sudden light coma with repeated generalized tonic-clonic seizures, with the most extended duration lasting for about 90 s. He was transferred to the neurological ICU for treatment with a GCS score of 5. Twenty-four-hour bedside quantitative EEG monitoring was immediately performed, which showed simultaneous epileptic discharges in both cerebral hemispheres during convulsive seizures. Sodium valproate 1,200 mg QD (from July 7th to July 10th), and Oxcarbazepine 300 mg Q12H combined Levetiracetam 1 g Q12H (from July 9th to August 16th) were then used. The epilepsy did not recur.

On July 8th, a lumbar puncture was performed to measure the initial pressure of 210 cmH_2_O and the final pressure of 135 cmH_2_O. CSF was collected and submitted for examination. Biochemical combination of CSF revealed glucose of 3.0 mmol/L, CSF chloride (Cl) of 131 mmol/L, CSF protein (PROT) of 734.7 mg/L, synchronous blood glucose of 5.9 mmol/L, and blood chloride of 117 mmol/L.

Samples were also sent for PACEseq mNGS (Hugobiotech, Beijing, China). QIAamp DNA Micro Kit (QIAGEN, Germany) was used for DNA extraction, and the libraries were then constructed using QIAseq™ Ultralow Input Library Kit for Illumina (QIAGEN, Germany). The quality of libraries was assessed by Qubit (Thermo Fisher) and Agilent 2100 Bioanalyzer (Agilent Technologies). The qualified DNA libraries were finally sequenced on Nextseq 550 platform (Illumina). Adapter, short, low-quality, and low-complexity reads were removed from the raw data. The human DNA was filtered out by mapping to human reference database (hg38). The remaining reads were finally aligned to the Microbial Genome Databases (http://ftp.ncbi.nlm.nih.gov/genomes/). B19V DNA (2 specific reads) in CSF was detected. Polymerase chain reaction (PCR) was then performed using B19V Q-PCR Detection Kit (ZJ Bio-Tech, Shanghai, China) and confirmed the mNGS results. CSF cytology showed a lymphocyte-monocyte reaction pattern. CSF routine, bacterial culture, autoimmune encephalitis antibody detection, and oligoclonal bands (OB) were unremarkable. Determination of CSF B19V DNA on July 9th showed 1.0 × 10^∧^ copies/mL. B19V caused encephalitis was considered.

After transfer, the patient's pneumonia was gradually aggravated, with type I respiratory failure and oxygenation index <200 on July 11th. Chest radiography showed inflammatory infiltration in bilateral lungs with bilateral pleural effusion. Brain plain MRI + enhancement MRI + DWI showed only scattered ischemic lesions in bilateral cerebral hemispheres. Echocardiography and color Doppler ultrasound of liver, gallbladder, pancreas, and spleen were unremarkable. Determination of B19V DNA in blood on July 11st showed 2.96 × 10^5^ copies/mL; B19V DNA and *Enterococcus faecium* was detected by PACEseq mNGS on July 13th, with the specific reads number of 1,430 and 22, respectively. Blood B19V IgM was negative, and IgG was positive. The reexamination of EEG on July 14th showed no epileptic discharges. Determination of B19V DNA in pleural fluid on July 15th showed the DNA determination of 1.36 × 10^5^ copies/mL; mNGS detected B19V in BALF on July 15th, with the specific reads number of 11, *Corynebacterium striatum*, with the particular reads number of 250, *Acinetobacter baumannii*, with the particular reads number of 17, *Enterococcus faecium*, with the specific reads number of 13, *Staphylococcus aureus*, with the particular reads number of 11, *Pseudomonas aeruginosa*, with the particular reads number of 11. In addition to the symptoms of the central nervous system, the patient's anemia was progressively aggravated. After multiple blood transfusions and platelet therapy, the lowest hemoglobin was 58 g/L on August 8th, the proportion of reticulocytes was 0.0026, the reticulocyte count was 0.0089 × 10^12^/L, the lowest platelet count was 60 × 10^9^/L, red blood cells and platelets were inhibited, and red blood cell morphology examination showed shrinkage and acanthocytes. The patient was finally diagnosed with encephalitis caused by B19V, secondary epilepsy, severe anemia, and bilateral pneumonia.

**Figure 2:**
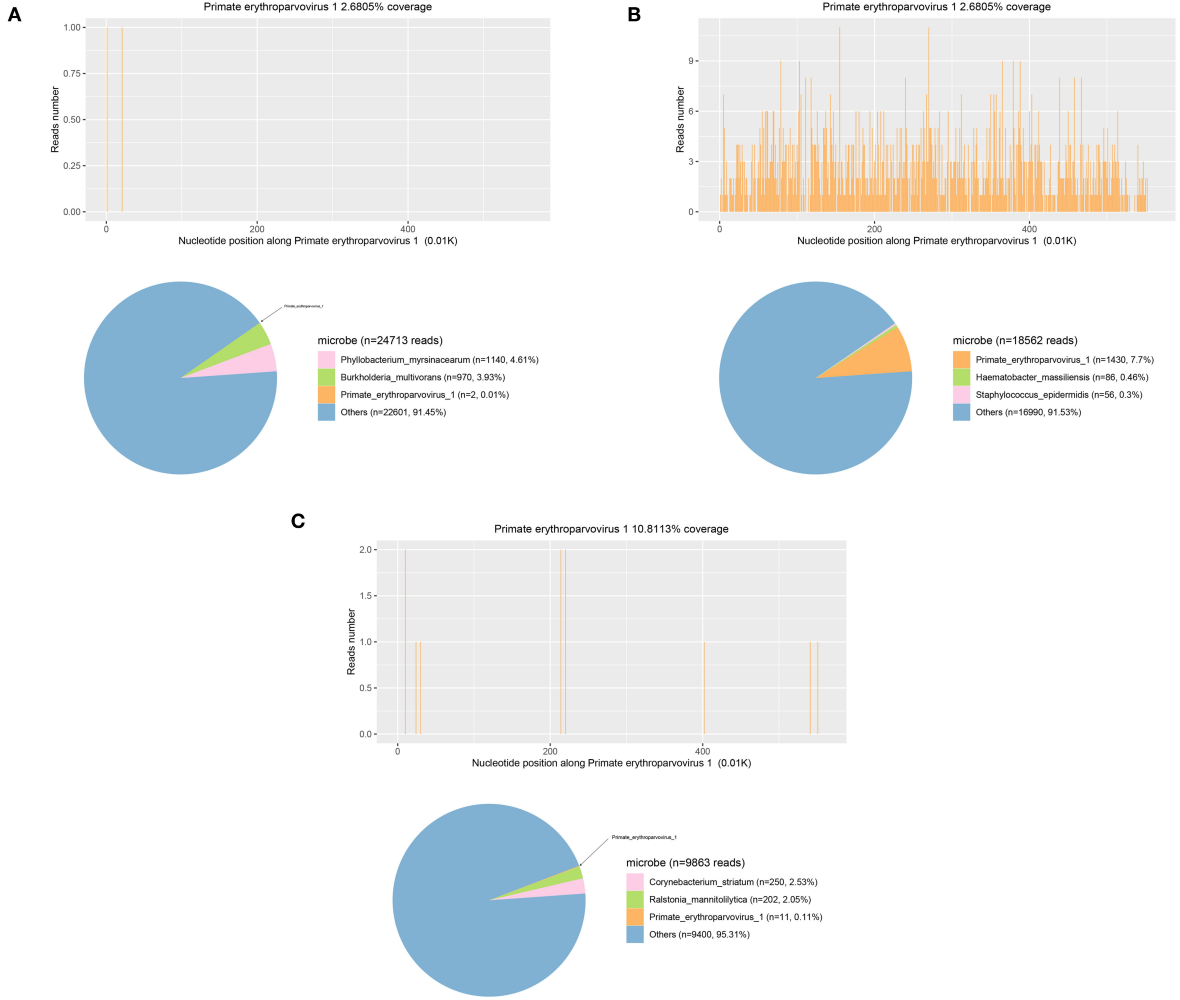
mNGS detection of CSF **(A)**, blood **(B)**, and bronchoalveolar lavage fluid samples **(C)**.

**Figure 3:**
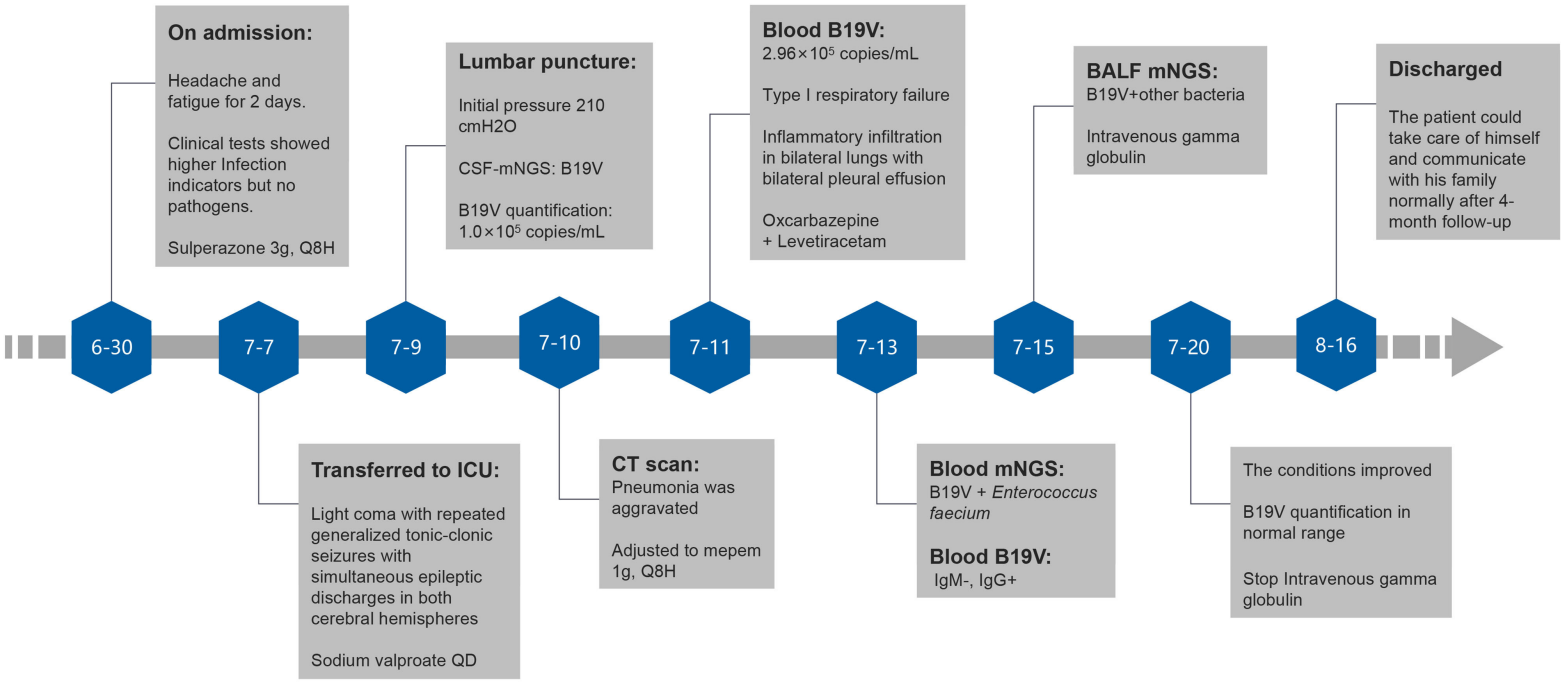
The timeline of this case.

The patient was given mepem 1 g, Q8H on July 10th, and then switched to vancomycin 1 g, Q12H on July 18th, intravenous gamma globulin 0.4 g/Kg body weight for 5 days from July 15th for symptomatic treatment of heart failure, renal failure, and other symptoms of organ damage. The patient did not have any more seizures. He gradually became conscious, opened and closed his eyes when instructed, made eye contact when called, GCS score increased to 10 points, oxygenation index was more significant than 300, and anemia was improved. On July 20th, reexamination showed the proportion of reticulocytes was 0.0702, and reticulocyte count was 0.2078 × 10^12^/L. On July 27th, reexamination showed that blood B19V DNA was <1,000 copies/ml. The number of mNGS specific reads and viral DNA quantification in the different specimens are showed in Table 1, while mNGS outcomes belong to the patient are showed in Figure 2. Afterment treatment, the patient had clear consciousness and could simply communicate with his family. He was finally discharged on August 16th. There was no sequelae or relapse of this patient after 4-month follow-up. The patient could take care of himself and communicate with his family normally. The timeline of this case was shown in Figure 3.

**Table 1:** Test results of PV B19 in various body fluid samples of the patient.

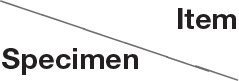	**Number of mNGS specific reads (time of collection)**	**Viral DNA quantification (copies/ml) (time of collection)**
CSF	+, 2 (2021-7-8)	1.0 × 10^∧^5 (2021-7-8)
Serum 1	+, 1,430 (2021-7-13)	2.96 × 10^∧^5 (2021-7-11)
Hydrothorax	–	1.36 × 10^∧^5 (2021-7-15)
BALF	+, 11 (2021-7-15)	/
Serum 2	Not reexamined	<1,000 (2021-7-27)

## Discussion and Conclusion

Human parvovirus infection mainly affects the skin, respiratory system, joints, and hematologic system, characterized by rash, flu-like symptoms, self-limiting arthritis, and transient erythroid hematopoietic disorders (17–19), the clinical manifestations of which lack specificity. In addition, it is difficult to isolate the virus by conventional cell culture, hence not feasible in clinical practice (20). Therefore, its diagnosis relies on serology and DNA detection (21). At the same time, real-time qPCR has been proven to be more reliable, especially in the diagnosis of recurrent or persistent infections, as antibody production may be lacking or minimal in these individuals (21): B19V IgM appears 1 week after infection (22) and persists for 2–3 months (21, 22). Due to tremendous differences between individuals, parvovirus B19V IgM may not be detected. IgG can persist after infection. A 4-fold increase of titer in 2 weeks is usually used to diagnose B19V infection. However, we did not test the titer after 2 weeks due to the use of gamma globulin treatment.

In this case, blood IgM test results were negative. Still, B19V DNA was detected in multiple serum samples, CSF, pleural fluid, and BALF. Though the detected DNA in blood might also be non-infectious naked DNA sipping out from the injured tissues, the high titer indicated that the DNA detected by real-time PCR is more likely from replicating virus. In addition, the discovery of B19V DNA and various bacteria in BALF can confirm that it is at least partly directly related to pneumonia (23–25). Due to convulsions during the course of the disease, lumbar puncture, and CSF mNGS detection also revealed B19V. The patient's CSF routine, biochemistry, protein, and cytology tests showed viral encephalitis changes, consistent with the diagnosis of B19V encephalitis (12).

On the other hand, after admission, the patient had a progressive decrease in hemoglobin and severe anemia, which was not improved significantly after multiple transfusion therapy. Reticulocyte level was extremely low, along with thrombocytopenia and leukopenia. Although B19V can inhibit erythroid proliferation, but severe anemia rarely occurs in immunocompetent patients with B19V infection. Immunocompromised patients with B19V infection are more likely to have decreased levels of erythrocytes and platelets or even whole blood cells (26). In this case, the blood routine showed severe immunosuppression, explaining the suppression of red blood cells and platelets.

Unexplained liver function impairment with B19V infection may be associated with the direct action of the virus, with elevated trans-aminases as the primary manifestation, which may be clinically asymptomatic or accompanied by acute hepatitis, primarily seen in children (27–30). Although the patient had liver function impairment mainly shown by elevated bilirubin, his liver enzymes were normal, so it could not rule out that drugs were the cause.

The majority of existing reports of B19V infection occur in children, pregnant women, and immunosuppressed populations. In contrast, infection accounts in immunocompetent individuals are relatively rare, and the symptoms are mild or asymptomatic. This case is a middle-aged male with acute onset and severe condition, not the ordinary population of previous B19V cases. He had no long-term immunosuppression, history of exposure to chemicals, poisons, animals, or other underlying conditions that can cause a state of immunosuppression. Meanwhile, there are few B19V involving multiple systems and accompanied by multiple organ failure (12, 23). The possibility of B19V infection is rarely considered in routine pathogen screening.

However, when evaluating the immune function of patients, it should also be taken into account that, in addition to organ transplantation, AIDS, long-term use of immunosuppressive drugs or drug abuse, severe infection might also cause suppressed humoral and cellular immune status and lead to immunosuppression, especially in clinical settings. The immune-related indicators of the patient suggested severe immunosuppression, with low lymphocyte count and proportion, mainly B lymphocytes, which might be due to latent viral infection after the last sepsis-induced immunosuppressive state of more than 2 months ago.

The presence of high-titer B19V was eventually identified with the aid of mNGS technology and subsequently confirmed in multiple clinical samples. The manifestation of multisystem involvement due to B19V infection was resolved with the addition of targeted therapy, so it was considered a multi-organ infection caused by B19V. The outbreak of B19V infection might be due to the severe immunosuppression caused by sepsis 2 months before.

The second-generation sequencing technology is significant for detecting rare or unexpected pathogens. It is an essential auxiliary means for the etiological diagnosis of patients with severe infections, which should be timely applied in the diagnosis and treatment process. However, it should be noted that the interpretation of mNGS results should be comprehensive and careful. This case shows that although mNGS has a more substantial pathogen detection capability than conventional detection, the number of virus-specific reads does not match the DNA quantification, resulting in a small number of mNGS reads. While the viral load is large and the clinical symptoms are severe. Therefore, the etiological hints of mNGS should be used rationally, and its results should not be based solely on its reads number, nor should mNGS be a substitute for quantitative pathogen detection.

Currently, there are no specific antiviral drugs for B19V infection. Treatment options depend on host factors such as immune status, potential underlying diseases, and manifestations of infection (31). Due to the short duration of symptoms, most infections in immunocompetent hosts do not require treatment. Intravenous gamma globulin (IVIG) 0.4 g/Kg bodyweight for 5 consecutive days can effectively treat a variety of clinical symptoms caused by B19V that could not recover spontaneously (32–35). However, the treatment is not once and for all, and in individuals with severe anemia treated with IVIG, the anemia may recur once the passive antibody effect is diminished (36, 37). If the hematocrit begins to decline, the B19V viral load should be measured again to identify the cause. The next IVIG course can be performed depending on the degree of anemia (20, 38, 39).

It should be noted that in the absence of symptoms, even after IVIG treatment, B19V DNA in serum can be detected by polymerase chain reaction (PCR) at low levels over several months to years (21, 40–43). Therefore, the evaluation of the therapeutic effect should be combined with clinical practice. PCR positive results alone should not be an indication for treatment, nor should the elimination of B19V DNA from the blood be the goal of therapy. After treatment with gamma globulin, the reticulocyte count increased, hemoglobin level increased, and serum viral nucleic acid load reduced significantly. The function of various organs was improved, and the patient was discharged, confirming the effectiveness of the treatment regimen. *Enterococcus faecium* was detected by mNGS in both blood and BALF samples. Though multiple anti-bacteria drugs were used for treatment, the conditions did not improve. However, the bacteria infection especially in the lung could not be ruled out.

In addition, corticosteroids can be used for central nervous system symptoms caused by B19V infection. However, there is still a lack of objective evaluation in the efficacy of IVIG and corticosteroids or a combination of the two (12). In addition, although cyclosporine has been reported as the treatment of B19V (44), it is usually recommended to reduce the use of immunosuppressive agents to facilitate the body's immune system to be activated and neutralize the virus (45). For other symptoms caused by B19V, such as rash and arthritis, most of them subside spontaneously, and non-steroidal anti-inflammatory drugs may be helpful for arthropathy (46). Patients with a transient aplastic crisis may require transfusion support (47).

This case's diagnosis and treatment experience have contributed to our understanding of the etiological detection of infectious diseases. The clinical manifestations caused by B19V infection are diverse. Still, few reports involve multiple organ or even concurrent central nervous system infection (23), and the rarity of B19V infection brings difficulties in diagnosis. Clinicians should pay attention to the immune status of patients in the disease state and rationally use mNGS technology to assist clinical diagnosis.

## Data Availability Statement

The original contributions presented in the study are included in the article/supplementary materials, further inquiries can be directed to the corresponding author/s.

## Ethics Statement

The studies involving human participants were reviewed and approved by the Ethics Committee for Clinical Research and Animal Trials of the First Affiliated Hospital of Sun Yat-sen University (Guangzhou, China). The patients/participants provided their written informed consent to participate in this study.

## Author Contributions

QZ, PC, and LF participated in collecting data and drafted the manuscript. JC and DC collected the data for case presentation. HX analyzed the mNGS data. LC participated in clinical management. HF and LF reviewed the literature and participated in its design. All authors read and approved the final manuscript.

## Funding

This study was supported by grants from Guangdong Provincial Key Laboratory of Diagnosis and Treatment of Major Neurological Diseases (2020B1212060017), Guangdong Provincial Clinical Research Center for Neurological Diseases (2020B1111170002), the Southern China International Cooperation Base for Early Intervention and Functional Rehabilitation of Neurological Diseases (2015B050501003 and 2020A0505020004), Guangdong Provincial Engineering Center For Major Neurological Disease Treatment, Guangdong Provincial Translational Medicine Innovation Platform for Diagnosis and Treatment of Major Neurological Disease, and the Clinical Research Supporting Programe for Lingnan Junior Doctors of Guangdong Province Medical Association (2021067).

## Conflict of Interest

HX was employed by Hugobiotech Co., Ltd. The remaining authors declare that the research was conducted in the absence of any commercial or financial relationships that could be conducted as a potential conflict of interest.

## Publisher's Note

All claims expressed in this article are solely those of the authors and do not necessarily represent those of their affiliated organizations, or those of the publisher, the editors and the reviewers. Any product that may be evaluated in this article, or claim that may be made by its manufacturer, is not guaranteed or endorsed by the publisher.
